# Quantifying the interrelationships between physical, social, and cognitive-emotional components of mental fitness using digital technology

**DOI:** 10.1038/s44184-024-00078-7

**Published:** 2024-07-08

**Authors:** Frank Iorfino, Mathew Varidel, William Capon, Matthew Richards, Jacob J. Crouse, Haley M. LaMonica, Shin Ho Park, Sarah Piper, Yun Ju Christine Song, Carla Gorban, Elizabeth M. Scott, Ian B. Hickie

**Affiliations:** Brain and Mind Centre, The University of Sydney, Sydney, Australia

**Keywords:** Disease prevention, Public health, Quality of life, Risk factors

## Abstract

Mental fitness is a construct that goes beyond a simple focus on subjective emotional wellbeing to encompass more broadly our ability to think, feel, and act to achieve what we want in our daily lives. The measurement and monitoring of multiple (often interacting) domains is crucial to gain a holistic and complete insight into an individual’s mental fitness. We aimed to demonstrate the capability of a new mobile app to characterise the mental fitness of a general population of Australians and to quantify the interrelationships among different domains of mental fitness. Cross-sectional data were collected from 4901 adults from the general population of Australians engaged in work or education who used a mobile app (Innowell) between September 2021 and November 2022. Individuals completed a baseline questionnaire comprised of 26 questions across seven domains of mental fitness (i.e., physical activity, sleep and circadian rhythms, nutrition, substance use, daily activities, social connection, psychological distress). Network analysis was applied at both a domain-level (e.g., 7 nodes representing each cluster of items) and an individual item-level (i.e., 26 nodes representing all questionnaire items). Only 612 people (12%) were functioning well across all domains. One quarter (*n* = 1204, 25%) had only one problem domain and most (*n* = 3085, 63%) had multiple problem domains. The two most problematic domains were physical activity (*n* = 2631, 54%) and social connection (*n* = 2151, 44%), followed closely by daily activity (*n* = 1914, 39%). At the domain-level, the strongest association emerged between psychological distress and daily activity (*r* = 0.301). Psychological distress was the most central node in the network (as measured by strength and expected influence), followed closely by daily activity, sleep and circadian rhythms and then social connection. The item-level network revealed that the nodes with the highest centrality in the network were: hopelessness, depression, functional impairment, effortfulness, subjective energy, worthlessness, and social connectedness. Social connection, sleep and circadian rhythms, and daily activities may be critical targets for intervention due to their widespread associations in the overall network. While psychological distress was not among the most common problems, its centrality may indicate its importance for indicated prevention and early intervention. We showcase the capability of a new mobile app to monitor mental fitness and identify the interrelationships among multiple domains, which may help people develop more personalised insights and approaches.

## Introduction

Mental health is a fundamental part of our overall health and wellbeing, and it is commonly recognised that there is “*no health without mental health”*^1^. This sentiment reflects how mental health is not just isolated to cognitions and emotions (how we think and feel), but instead it connects to many social and biological components of what it means to be human. This broad view of mental health encapsulates the related but distinct concepts of mental illness and mental fitness.

Mental fitness is a continuum that encompasses personal autonomy alongside social connection and participation. It goes beyond a simple focus on ‘mood’ or subjective emotional wellbeing to encompass more broadly our ability to think, feel, and act to achieve what we want in our daily lives. It is not a new concept and many versions of it have been presented over the past few decades which includes concepts of positive psychology, wellbeing, and flourishing^2,3^. The pursuit of a mental fitness concept largely reflects the need for a construct that is measurable, improvable (like physical fitness), and distinct from mental illness in that it focuses on the positive aspects of human cognition, emotion, and behaviour^4^. By contrast, mental illness (or disorder), which has a distinct focus on distressing experiences and symptoms, has its own continuum of illness stages and severity that have differential risks of progression and recurrence^5,6,7^.

**Fig. 1:**
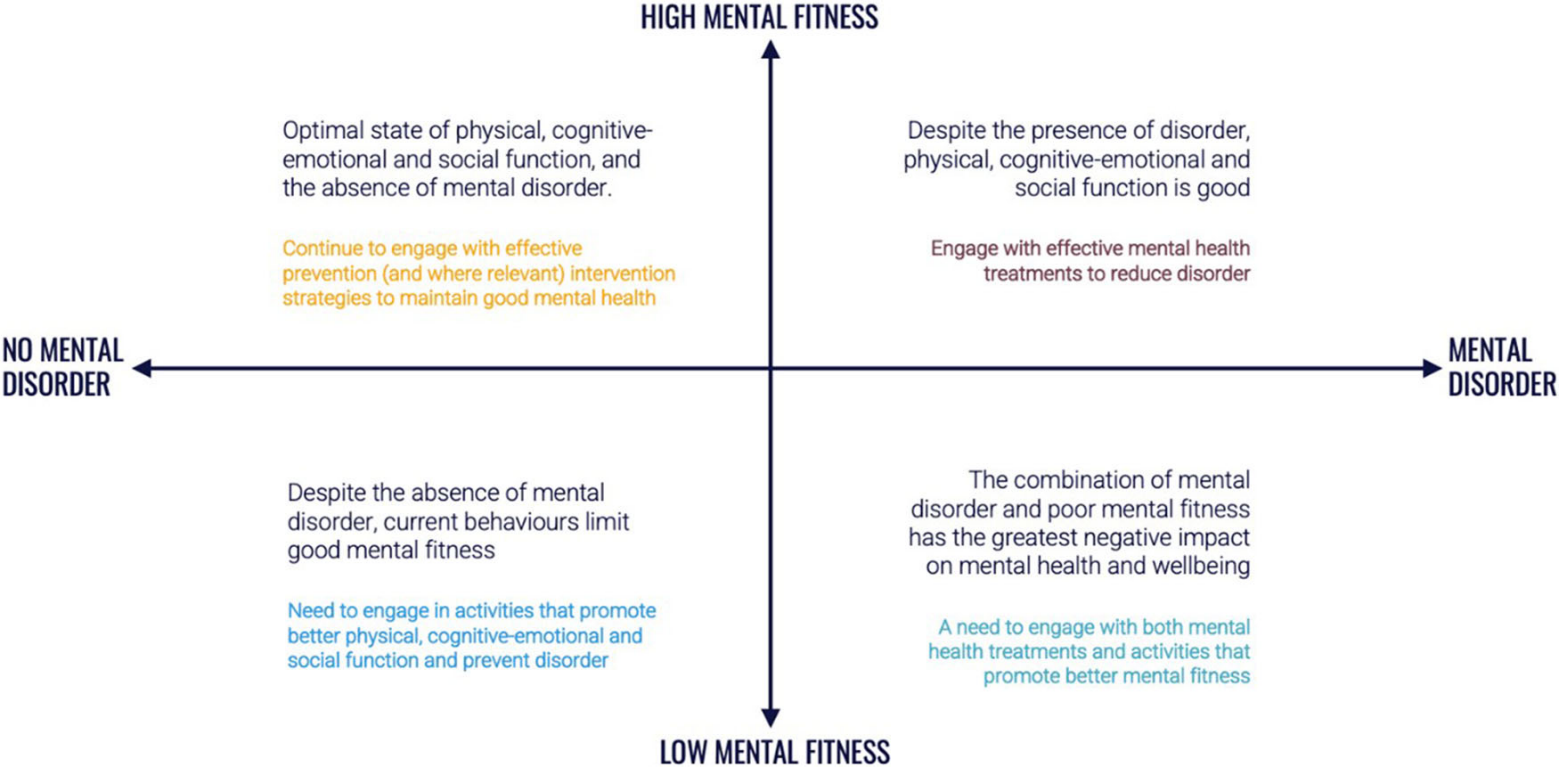
The continuums of mental fitness and the mental disorder.

Globally, it is estimated that almost one in three people will experience a mental disorder in their lifetime, and that approximately one in five people have experienced a major disorder within the past 12-months^8^. Many people may never experience a mental illness, though all of us will move up and down the related but distinct continuum of mental fitness over the course of our lives (Fig. 1). For some, these oscillations may be more frequent or severe, and increase the risk of more serious mental health problems and illness. For others, the distinction between illness and fitness reflects how functioning and mental health may not always be directly correlated, particularly for those self-managing an illness (e.g., those in the top right quadrant of Fig. 1). Much like we do for our physical health, using evidence-based strategies to monitor and enhance mental fitness, alongside regular checks of our mental health, is important to promote to the widest possible population groups.

A focus on multiple (often interacting) domains is important to gain a holistic and complete insight into an individual’s mental fitness. Current holistic measures tend to focus on subjective positive well-being states and quality of life based on positive psychology concepts, though few measures capture a broader set of behaviours important for mental fitness^9,10^, including those that emphasize physiological and not just psychological domains. The domains most relevant for this include aspects of our physical health related to physical activity, sleep and circadian rhythms, nutrition and substance use; our cognitive-emotional health (e.g., mood, anxiety, stress); and our social health, which includes functioning in daily roles and social connections^11–21^. Problems in any one of these domains may be indicative of an increased risk for poorer health outcomes that may be due to dysfunction (or sub-optimal function) in one or more underlying systems which govern most of our behaviour, cognition, and emotion. For example, poor cognitive-emotional health could reflect prolonged activation of the stress response system^22^, which regulates our hormonal response to stress; or poor sleep quality or energy during the day may suggest dysfunction of the circadian system, which orchestrates the daily rhythmic timing of almost all physiological processes and behaviours^23^.

The rise of digital technologies provides the opportunity for monitoring and managing various aspects of our mental fitness. While there are numerous health apps available^24^, there is a growing need for more comprehensive tools that can assess and address the interconnected nature of these social, physical and cognitive-emotional dimensions. By identifying the patterns and relationships between these dimensions, people can develop more personalised insights and recommendations to enhance their unique mental fitness and reduce future risks. The objective of this paper is to demonstrate the capability of a new mobile app (‘Innowell’) to characterise the mental fitness of a general population of Australians and to quantify the interrelationships among the social, physical, and cognitive-emotional domains of mental fitness.

## Results

### Domain Ratings

**Fig. 2:**
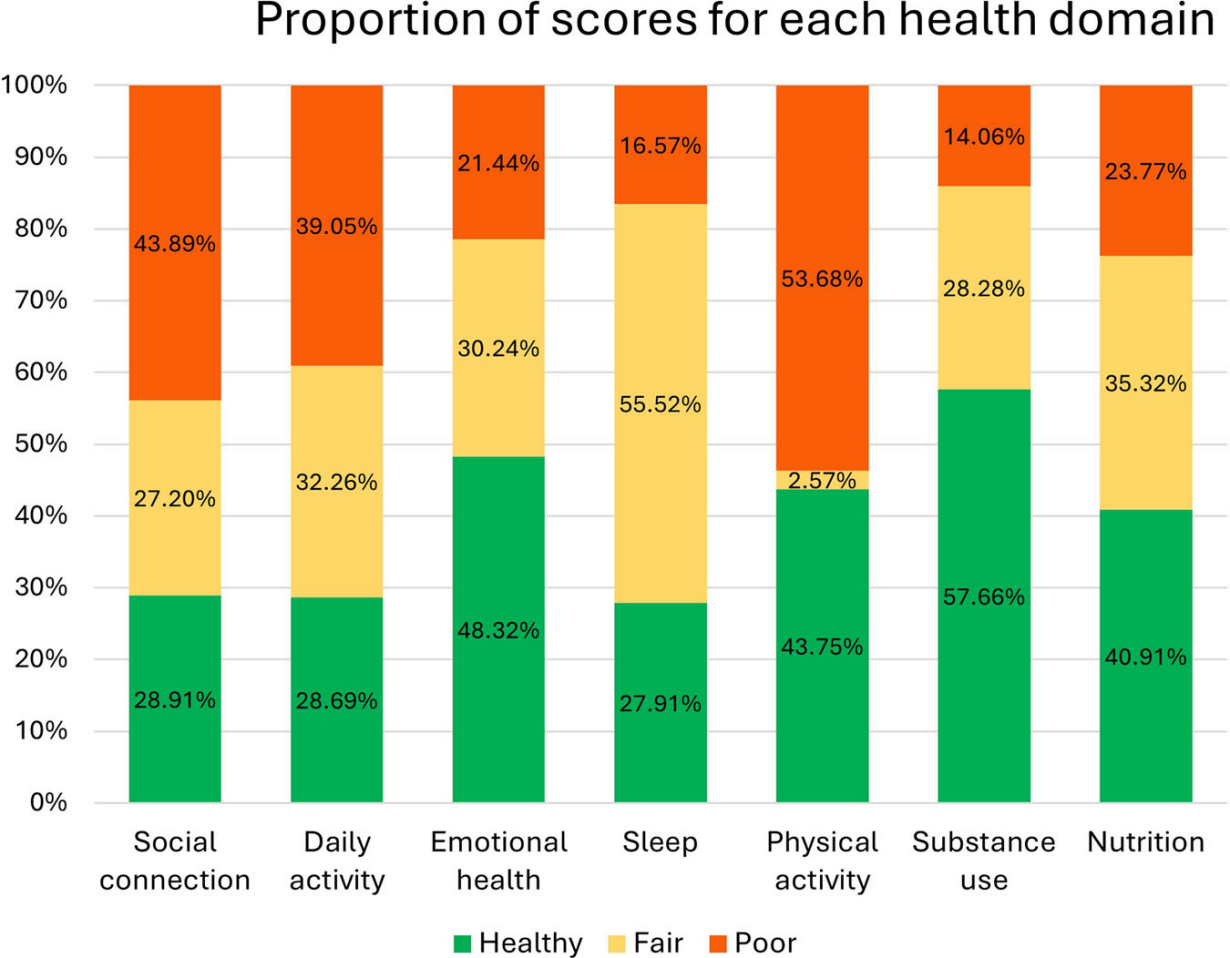
Proportion of scores for each health domain. Total completions n = 4901.

A total of 4901 individuals completed the initial questionnaire and were eligible for analyses, though socio-demographics were not available for this study. Only 612 people (12%) of the population had scores that were ‘healthy’ or ‘fair’ across all domains. About a quarter (*n* = 1204, 25%) had only one ‘poor’ domain, and the majority (*n* = 3085, 62%) had multiple ‘poor’ domain. The two most problematic domains were physical activity (*n* = 2631, 54%) and social connection (n = 2151, 44%), followed closely by daily activity (*n* = 1914, 39%) (Fig. 2).

### Domain-level Network

**Fig. 3:**
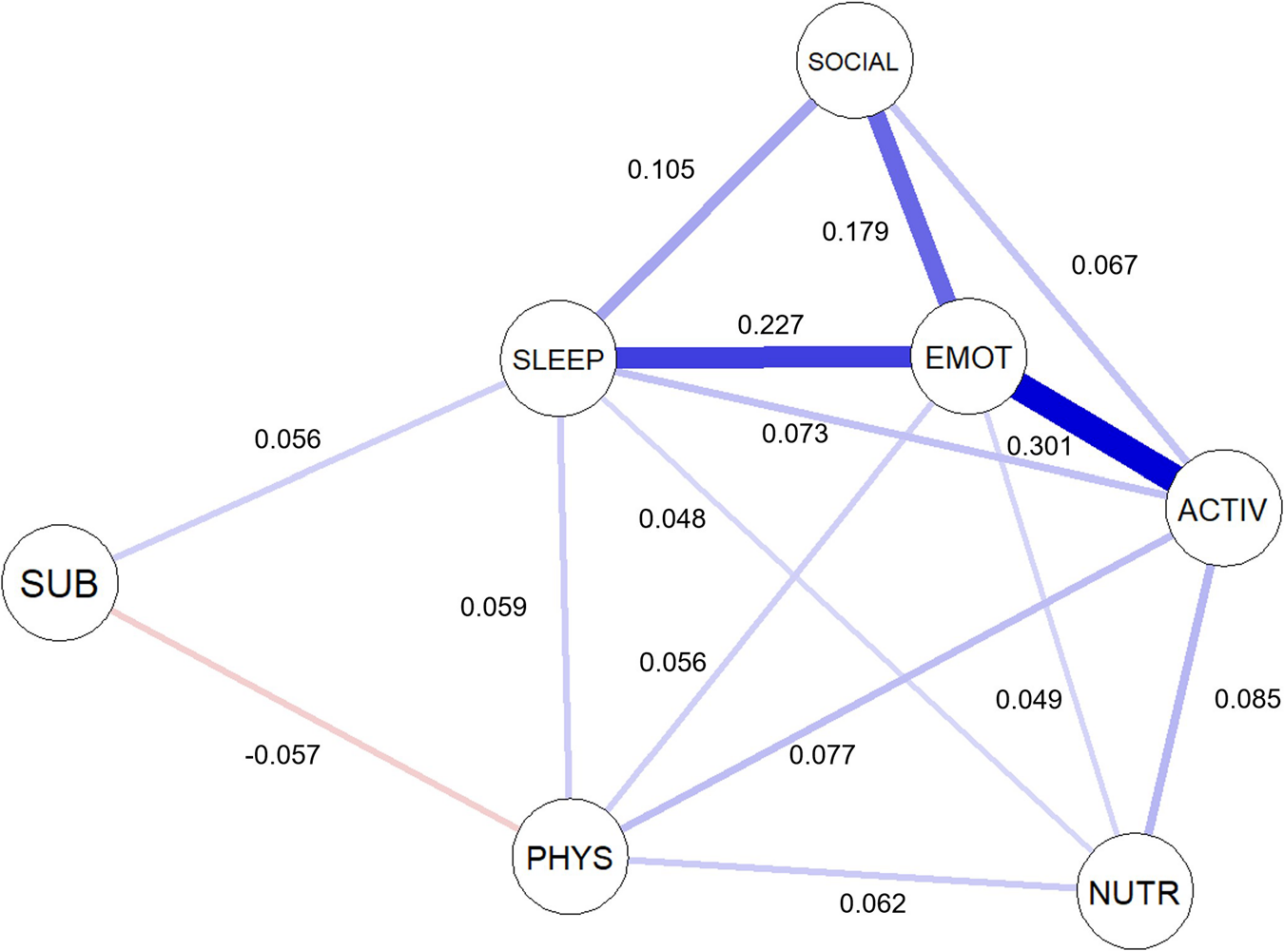
A network model to infer cross-sectional relationships between social, cognitive-emotional, and physical health areas for a general population (*n* = 4901). The network plot was developed in accordance with the Fruchterman-Reingold algorithm^60^, which places edges with greater absolute coefficient values closer together. The blue edges represent positive partial correlations, whereas the red edges represent negative partial correlations. Social = social connection; activ = daily activities; emot = psychological distress; sleep = sleep and circadian rhythms; phys = physical activity; sub = substance use; and nutr = nutrition.

**Fig. 4:**
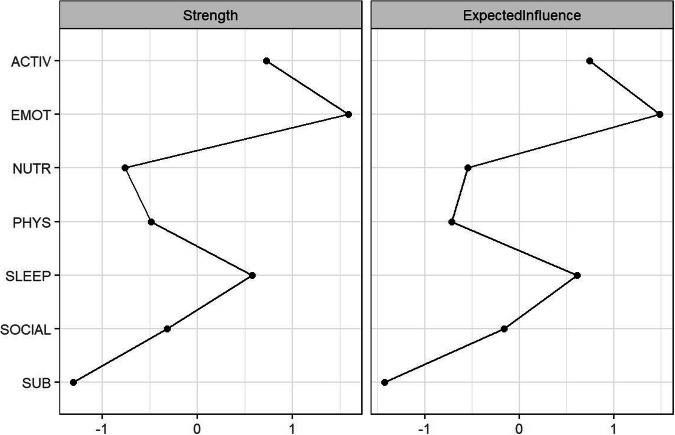
Domain-level centrality plots. Social = social connection; activ = daily activities; emot = psychological distress; sleep = sleep and circadian rhythms; phys = physical activity; sub = substance use; and nutr = nutrition.

The domain-level network is presented in Fig. 3. Each domain was connected either directly or indirectly via other domains in the network. The strongest association emerged between psychological distress and daily activity (*r* = 0.301), while the weakest non-zero association was between nutrition and sleep and circadian rhythms (*r* = 0.048). Of the possible 21 associations across all domains, 71.43% (*n* = 15) were nonzero with only one negative relationship identified, between substance use and physical activity (*r* = −0.057). Psychological distress was the most central node in the network (as measured by strength and expected influence) (Fig. 4), followed closely by daily activity and sleep and circadian rhythms.

### Item-level Network

**Fig. 5:**
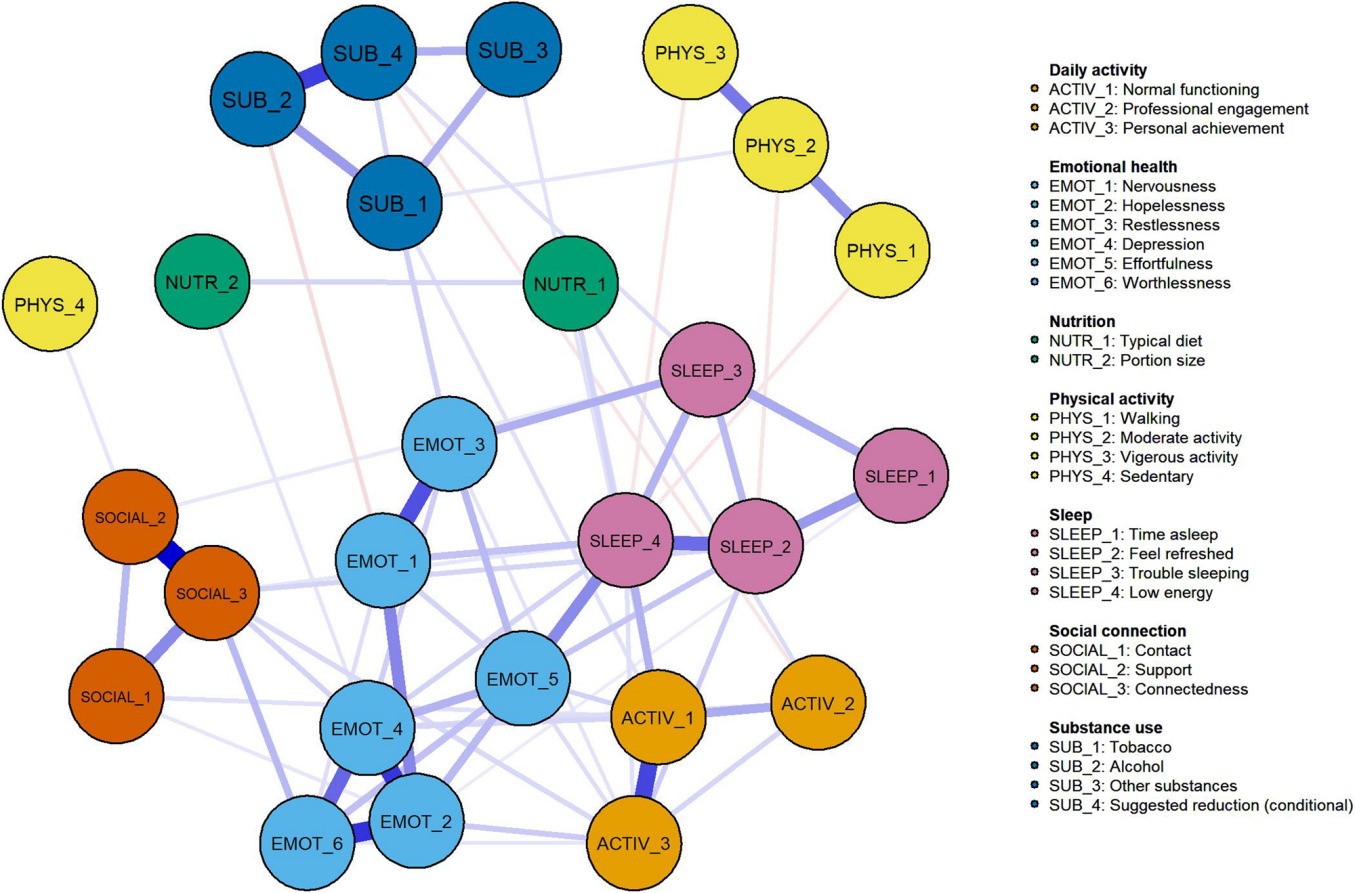
Item-level network model to infer cross-sectional relationships between 26 items from the Innowell Questionnaire.

**Fig. 6:**
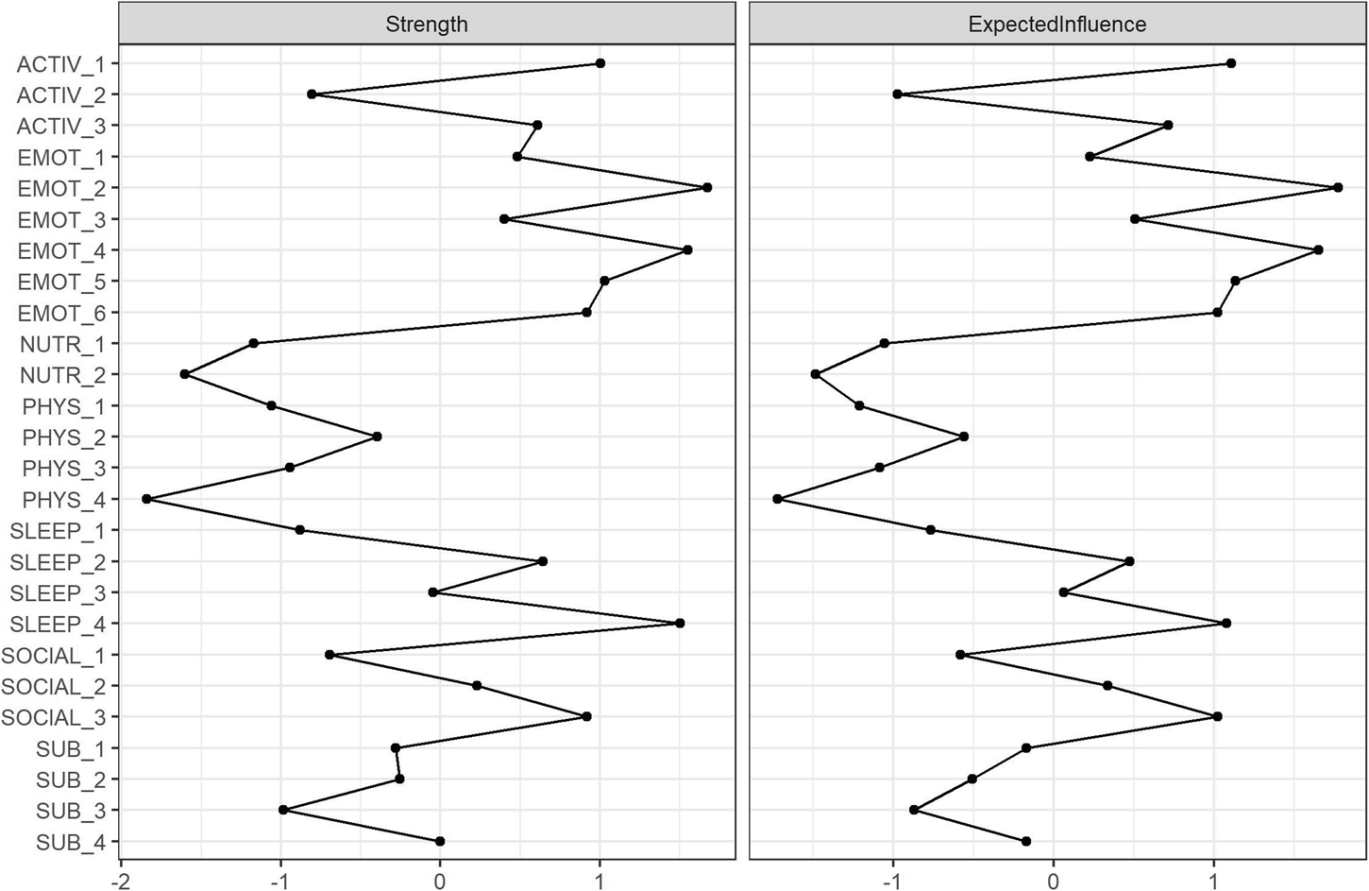
Item-level centrality plots.

For the item-level network, all items were connected either directly or indirectly via other symptoms in the network (Fig. 5). The nodes with the highest expected influence centrality in the network were (Fig. 6); Emot_2 (Hopelessness), Emot_4 (Depression), Activ_1 (Normal functioning), Emot_5 (Effortfulness), Sleep_4 (Subjective energy), Emot_6 (Worthlessness), and Social_3 (Connectedness).

## Discussion

This study presents cross-sectional data about the mental fitness of a general population of adult Australians and quantifies the relationship between these domains. Very few people were healthy across all domains, with most of the population having two or more areas that could be improved. Physical activity, social connection and daily activity were the three most common problem areas. Centrality analyses indicated that psychological distress, daily activity, and sleep and circadian rhythms had the highest influence in the network. This work highlights which domains may be particularly important for mental fitness, however longitudinal studies of these relationships would offer further insights about indicated prevention and early intervention approaches.

The psychological distress domain asks people about common symptoms of depression, anxiety, and stress. Four individual items from this domain (hopelessness, depression, effortfulness, and worthlessness) were part of the top five most central nodes in the item-level network. In conjunction with psychological distress being the most central node in the domain-level network, this finding demonstrates how cognitive-emotional health is strongly linked to both social and physical health. While psychological distress is increasingly common in Australia’s general population^25^, it is positively associated with mental ill-health^18,19^.

In this cohort, only 21% were identified as having ‘poor’ psychological distress, yet its strong connections with common problem areas (social connection, sleep and circadian rhythms, and daily activity) emphasise the need for indicated prevention and early intervention in these areas to avoid the development of vicious cycles of worsening outcomes. The strongest domain-level association was between psychological distress and daily activity. Daily activity specifically measures whether a person can ‘carry out daily tasks’ and ‘achieve the things you want to’ in daily roles, and so its widespread impact to other areas is significant. Functional impairment is common among those with mental illness and changes in functioning are often an early sign of mental ill-health^18,26–29^. Major functional impairment is associated with worse mental health outcomes^30^, future suicidality^31^, and is positively correlated with the severity of mental illness^7^. This reinforces the need for regular monitoring of daily activities and functioning, so that early changes or problems in this area can be promptly detected and proactively responded to before these problems get worse or exacerbate problems in other areas.

Social connection was one of the most commonly reported problems with high centrality, which is concerning given its known widespread effects on health and wellbeing. Social isolation and disconnection are associated with higher rates of mental disorders^32^, and associated with poor long-term outcomes (including increased mortality risk)^33^. We identified a strong association between social connection and psychological distress, specifically feelings of depression and worthlessness, which reiterates the increased vulnerability among socially isolated individuals. Growing evidence points to common mechanisms involving elevated activity to the sympathetic nervous system and hypothalamus-pituitary-adrenal axis, as well as chronic inflammation, which may link social isolation and disconnection to deteriorating mental health and other poor health outcomes^34,35^. Altogether, this work suggests that indicated prevention and early intervention strategies that help people develop and maintain quality social relationships with friends and family and foster deeper connection to their communities may be critical to enhance mental fitness and prevent further health problems in other areas^36^.

The centrality of sleep and circadian rhythms in the domain-network is particularly important given that it was identified as 'poor' or 'fair' for over two-thirds of the population and had the second strongest domain-level association (psychological distress and sleep and circadian rhythms, r = 0.227). The relationship between sleep and circadian rhythms and mental health is well established, with many people reporting a range of sleep and circadian disturbances (e.g. trouble falling asleep, insomnia, fatigue, etc)^12,37^ that are typically associated with increased stress, depression, and state anxiety, and usually worsen with more severe illness^7,38^. We identified that subjective energy had strong connections in the network, particularly with being able to ‘carry out daily tasks’ and ‘effortfulness’. This is consistent with evidence which indicates that subjective energy may be an effective intervention target for mood, as some targeted sleep and circadian interventions have been shown to also improve energy and mood^39,40^. Taking steps to improve the 24-hour sleep-wake cycle may contribute to better mood, cognitive-emotional regulation, help reduce symptoms of mental illnesses^39,41–43^, and may reduce the likelihood of developing a mental disorder^12,30^.

Despite the strong relationship between sleep and circadian rhythms with both social and cognitive-emotional health, the remaining areas of physical health (nutrition, physical activity, and substance use) had relatively weak associations with other domains in the network. Each of these domains tended to be quite independent. This may reflect that their emergence is dependent on other factors not measured by the current physical, social, and cognitive-emotional domains, and/or that their relationship with other domains is more specific to certain subgroups within this population, and therefore unable to be detected by the current analyses. Nonetheless, their independence could reflect that these physical health areas require more specific preventative and intervention strategies since they may be less amendable to cascading effects from improvements in other domains of social and cognitive-emotional health.

This study has several limitations that should be considered alongside our findings. Firstly, we did not collect demographics for the current analyses. Therefore, the interpretation and generalisability of these results is limited as we are unable to determine the effect of age, gender, and other characteristics on the networks or say anything about who is more likely to use this app. While this is a major limitation, the focus of this work was on the interrelationships between these key domains and so future work should aim to include more detailed analyses of these characteristics. Secondly, the data were cross-sectional which does not allow conclusions about the longitudinal relationship between domains. The interactions among these domains over time may have the greatest influence on mental fitness and the emergence of illness. Their effects are probably not simply additive, but are likely part of a complex system characterised by non-linear relationships and feedback loops, which could lead to unexpected outcomes^44^. Thus, to achieve a holistic view of a person’s mental fitness, it is not sufficient to focus on single components alone, but important to monitor how these factors evolve and influence each other over time. Future studies with time-series data are needed to determine this. Finally, we relied on self-report measures. There is a growing need for passive data collection which can reduce burden on participants, increase the accuracy of data collected, and provide high frequency and valuable insights about individual patterns over time.

In conclusion, most people have areas of mental fitness that could be improved. Social connection, sleep and circadian rhythms and daily activities emerged as being potential targets for intervention due to their widespread associations in the overall network. While psychological distress was not among the most common problems, it was highly central in the network with connections to all domains (except for substance use), emphasising the key role of psychological distress in overall health and wellbeing. We showcase the use of digital technologies to monitor mental fitness and identify the interrelationships among multiple domains, which may help people develop more personalised insights and recommendations to enhance their mental fitness and reduce future risks.

## Methods

### Ethics

All data for this study was collected through a quality assurance process facilitated by the University of Sydney research team. All data is non-identifiable to protect the privacy of participants.

### Innowell

Innowell is a mobile app that provides research validated, professional tools for people to monitor and reflect on their mental fitness. It includes (1) real-time assessments about each domain of a person’s mental fitness; (2) actionable insights about each domain of mental fitness; (3) personalised recommendations with evidence-based strategies and resources to understand and manage mental fitness; and (4) a goal setting and tracking tool which provide people with habit-forming activities designed to improve their mental fitness. The development of this tool involved a team of psychologists, psychiatrists, mental health research experts, and those with lived experience, who selected items that measure various components of mental fitness and collated evidence-based strategies and resources for mental fitness.

### Dataset

The dataset is comprised of adults from a general population of Australians engaged in work or education who used Innowell between September 2021 and November 2022. The platform was offered to them as part of a mental health and wellbeing initiative, and so sign up and completion of the baseline questionnaire was self-directed. All data has been anonymised and shared for quality assurance with no identifying personal information available for reporting, in accordance with the privacy policy. The only measures reported here are individual responses to real-time assessments for each of the mental fitness domains to understand the interrelationships between them.

The measures used for these domains include; (i) social connection, three items from Schuster’s Social Support Scale^45^ which ask about social contact, support and wider family, cultural or community connectedness; (ii) daily activities, three items which ask about educational and employment engagement and participation, and personal achievement^46,47^; (iii) psychological distress, six items from the Kessler-6 which ask about common symptoms of depression, anxiety and stress^48^; (iv) sleep and circadian rhythms, four items which ask about time spent sleeping, feeling refreshed after sleep, trouble falling asleep and subjective energy^49–51^; (v) physical activity, four items from the International Physical Activity Questionnaire to measure of time spent engaged in walking, moderate exercise, vigorous exercise and sedentary behaviours^52–55^; (vi) substance use, four items about the use of tobacco, alcohol and other substances, and whether they should cut down^56,57^; and (vii) nutrition, two items about typical diet type and portion size^58^. See Supplementary Table 1 for further details.

### Statistical analyses

All statistical analyses were performed in R version 4.2.1^59^.

Statistical analyses were conducted in R. Frequency counts and proportions were obtained for scores across each mental fitness domain. We then applied network analysis to the data at both a domain-level (e.g., 7-nodes representing each cluster of items), and an individual item-level (i.e., 26-nodes representing all questionnaire items) to identify the dependency structure between domains and items measuring mental fitness. For this a partial correlation network was estimated using the graphical Least Absolute Shrinkage and Selection Operator (gLASSO) algorithm^60^. This algorithm estimates the partial correlation coefficients using a regularisation technique that limits the number of spurious edges. The regularisation parameter for the gLASSO algorithm was selected by minimising the Extended Bayesian Information Criteria (EBIC)^61–64^. As the dataset contains ordinal data, we used the polychoric correlation matrix as input for the network estimation algorithm^61,63,64^.

Accuracy and stability of edge-weights in the network were examined using nonparametric and case-dropping subset bootstrapping procedures^62,64^. Strength and expected influence were considered as the most reliable centrality measures and both measures gave similar results. The strength represents the sum of the absolute partial correlation coefficients. Expected Influence uses the summed partial correlation coefficients (i.e., including the sign) extending away from each node. These measures estimate how central a node is in the diagram, which assuming relationships are bidirectional can be thought of as a measure of influence that changing that node will have on the other nodes. We report these centrality measures, along with their bootstrapped 95% intervals.

### Supplementary information

Supplementary Methods

## Data Availability

The data presented in this manuscript is available upon reasonable request.
