# Supplementary material for: Quantifying the interrelationships between physical, social, and cognitive-emotional components of mental fitness using digital technology
Source: Npj Ment Health Res. 2024 Jul 8;3:36. doi: 10.1038/s44184-024-00078-7 (PMC11231280; doi:10.1038/s44184-024-00078-7)
Supplement: Supplementary file 1 — Supplementary Methods [file 44184_2024_78_MOESM1_ESM.pdf]

Supplementary Methods

Supplementary Table 1. Questions and scoring for each domain.

Domain	Question	Answer Options
Sleep and circadian rhythms	How many hours of sleep did you usually get a night?	<= 6 hours
		6.5-7.5 hours
		8-10 hours
		10.5-11.5 hours
		>= 12 hours
	How often have you felt really refreshed when waking in the morning?	Never
		1 day/week
		2-3 days/week
		4-5 days/week
		6-7 days/week
	Did you often have trouble falling asleep (more than 30 minutes) or wake up frequently throughout the night (>= 3 times)?	Never
		1 day/week
		2-3 days/week
		4-5 days/week
		6-7 days/week
	How often did you feel that you had little or no energy during the day?	Never
		1 day/week
		2-3 days/week
		4-5 days/week
		6-7 days/week
Physical activity	Over the past week how much time did you spend walking?	User enters hours and minutes.
	Over the past week how much time did you spend doing moderate physical activities?	User enters hours and minutes.
	Over the past week how much time did you spend doing vigorous physical activities?	User enters hours and minutes.
	Over the past week how much time did you spend sitting or lying down on an average weekday?	User enters hours and minutes.
Social connection	When you think about the amount of contact you had with your friends and/or family, do you think it was:	Too much contact
		About the right amount of contact
		Not enough contact
	How often did your friends and/or family make you feel cared for?	Never
		Rarely
		Sometimes
		Often
		Strongly agree

	I felt a sense of mutual connection with my family, friends, community and culture.	Agree
		Neither agree nor disagree
		Disagree
		Strongly disagree
Functioning	How many days in total were you able to carry out your usual daily activities fully?	0
		1
		2
		3
		4
		5
		6
		7
	How many hours were you engaged in education, training, employment, or a designated carer role?	Wasn’t in education, training, employment, or a designated carer role
		Less than 5 hours
		5 to 14 hours
		15 to 29 hours
		30 hours or more
	How often did you achieve the things you wanted to?	Never
		Rarely
		Sometimes
		Often
Psychological distress	Over the past week, roughly how often did you feel nervous?	All of the time
		Most of the time
		Some of the time
		A little of the time
		None of the time
	Over the past week, roughly how often did you feel hopeless?	All of the time
		Most of the time
		Some of the time
		A little of the time
		None of the time
	Over the past week, roughly how often did you feel restless or fidgety?	All of the time
		Most of the time
		Some of the time
		A little of the time
		None of the time
		All of the time
		Most of the time

	Over the past week, roughly how often did you feel so depressed that nothing could cheer you up?	Some of the time
		A little of the time
		None of the time
	Over the past week, roughly how often did you feel that everything was an effort?	All of the time
		Most of the time
		Some of the time
		A little of the time
		None of the time
	Over the past week, roughly how often did you feel worthless?	All of the time
		Most of the time
		Some of the time
		A little of the time
		None of the time
Substance Use	How often have you used tobacco products?	Never
		Once or twice
		Daily or almost daily
	How often did you consume more than four standard drinks of alcohol on one occasion?	Never
		Once or twice
		Daily or almost daily
	How often have you used other substances?	Never
		Once or twice
		Daily or almost daily
	Have you considered (or has a friend, relative or someone else suggested) improving your health and wellbeing by reducing alcohol or substance consumption?	No, I don’t want or need to cut down
		Possibly, I’m thinking about it
		Yes, I’m currently trying to cut down
		A friend or relative or someone else has suggested it
Nutrition	Which of the following best describes your typical main meal?	Mostly vegetables, with some protein and carbohydrates
		An even mix of vegetables, protein, and carbohydrates
		Mostly carbohydrates, with some meat and vegetables

		Mostly meat, with some<br>vegetables and carbohydrates
		A mix of meat and carbs, with<br>little to no vegetables
	Which of the following pictures is closest to the serving size you usually consumed for a main meal?	Choice of serving sizes
